# Supplementary material for: Renin-angiotensin-aldosterone system variations in type 2 diabetes mellitus patients with different complications and treatments: Implications for glucose metabolism
Source: PLoS One. 2025 Mar 19;20(3):e0316049. doi: 10.1371/journal.pone.0316049 (PMC11922211; doi:10.1371/journal.pone.0316049)
Supplement: S1 Table — (DOCX) [file pone.0316049.s001.docx]

S1 Table. Antihypertensive treatment of 151 T2DM patients with hypertension at different subgroups.

| Antihypertensives | DN(n=31) | DK(n=20) | DNK(n=10) | OCHT(n=70) | NCHT(n=20) | Total(n=151) |
| --- | --- | --- | --- | --- | --- | --- |
|  | NO. (%) | NO. (%) | NO. (%) | NO. (%) | NO. (%) | NO. (%) |
| ACEI/ARBs | 27(87.1) | 14(70.0) | 8(80.0) | 47(67.1) | 14(70.0) | 110(72.8) |
| CCBs | 26(83.9) | 12(60.0) | 7(70.0) | 56(80.0) | 12(60.0) | 113(74.8) |
| β-Blockers | 12(38.7) | 3(15.0) | 3(30.0) | 24(34.3) | 3(15.0) | 45(29.8) |
| TDs | 6(19.3) | 3(15.0) | 1(10.0) | 10(14.3) | 1(5.0) | 21(13.9) |
| α- Blockers | 2(6.4) | 2(10.0) | 2(20.0) | 5(7.1) | 1(5.0) | 12(7.9) |
| Number of Antihypertensives |  | | | | | |
| 1 | 8(25.8) | 10(50.0) | 2(20.0) | 21(30.0) | 12(60.0) | 53(35.1) |
| 2 | 9(29.0) | 7(35.0) | 6(60.0) | 28(40.0) | 6(30.0) | 56(37.1) |
| 3 | 9(29.0) | 2(10.0) | 1(10.0) | 18(25.7) | 1(5.0) | 31(20.5) |
| 4 | 5(16.1) | 1(5.0) | 1(10.0) | 3(4.3) | 1(5.0) | 11(7.3) |

DN, diabetic nephropathy; DK, diabetic ketoacidosis; DNK, diabetic nephropathy with ketoacidosis; OCHT, other diabetic complications in hypertensive patients; NCHT, no complications in hypertensive patients; ACEI/ARBs, angiotensin-converting enzyme inhibitors/angiotensin receptor blockers; CCBs, calcium channel blockers; TDs, thiazide diuretics; NO., number.
