# Supplementary material for: Renin-angiotensin-aldosterone system variations in type 2 diabetes mellitus patients with different complications and treatments: Implications for glucose metabolism
Source: PLoS One. 2025 Mar 19;20(3):e0316049. doi: 10.1371/journal.pone.0316049 (PMC11922211; doi:10.1371/journal.pone.0316049)
Supplement: S2 Table — (DOCX) [file pone.0316049.s002.docx]

S2 Table. The influence of antihypertensive therapy on the RAAS system and glucose metabolism indexes.

| HT（n=151） | | | | | | | | | | |
| --- | --- | --- | --- | --- | --- | --- | --- | --- | --- | --- |
|  | ACEI/ARBs | | CCBs | | β-Blockers | | TDs | | α- Blockers | |
| Variables | No（41） | Yes（110） | No（38） | Yes（113） | No（106） | Yes（45） | No（130） | Yes（21） | No（139） | Yes（12） |
| HbA1c (%) | 10.11±1.92 | 9.35±2.20 | 10.00±2.30 | 9.41±2.09 | 9.53±2.09 | 9.62±2.32 | 9.47±2.01 | 10.11±2.90 | 9.64±2.14 | 8.61±2.16 |
| FBG (mmol/L) | 10.32±2.95 | 9.38±2.75 | 9.85±2.75 | 9.56±2.86 | 9.74±2.80 | 9.38±2.89 | 9.72±2.90 | 9.09±2.27 | 9.75±2.86 | 8.29±1.97 |
| AII (ng/L) | 112(105~128) | 116(106~125) | 113(102~121) | 116(106~127) | 114(105~124) | 118(107~127) | 114(106~124) | 120(107~129) | 114(106~124) | 129(120~134) * |
| ALD (ng/L) | 152(123~186) | 147(116~192) | 152(123~192) | 147(116~190) | 145(121~195) | 152(110~182) | 147(117~190) | 150(112~206) | 150(118~190) | 135(107~213) |
| REN (ng/L) | 12.0(5.4~25.4) | 12.3(6.2~26.8) | 15.2(7.6~30.3) | 11.7(5.7~24.7) | 13.0(7.3~30.0) | 11.0(3.9~17.8) * | 12.0(6.1~26.4) | 12.6(5.2~24.0) | 11.9(6.0~26.2) | 13.3(6.3~32.2) |
| ARR | 1.23(0.68~2.52) | 1.15(0.6~2.12) | 0.95(0.55~1.65) | 1.25(0.70~2.42) | 1.07(0.60~1.94) | 1.70(0.93~3.51) * | 1.20(0.63~2.14) | 1.18(0.54~3.41) | 1.20(0.63~2.30) | 1.15(0.54~1.95) |
| CP (nmol/L) | 0.65(0.42~0.96) | 0.73(0.46~1.02) | 0.80(0.55~0.99) | 0.67(0.41~1.02) | 0.66(0.41~1.02) | 0.74(0.52~0.98) | 0.67(0.43~1.02) | 0.79(0.42~1.00) | 0.70(0.45~1.02) | 0.65(0.32~1.13) |
| HOMA-β (%) | 38.6(21.9~62.6) | 41.9(28.4~59.8) | 41.4(28.0~58.8) | 41.6(26.4~62.6) | 39.4(26.5~58.8) | 45.6(27.4~70.8) | 40.0(26.5~59.8) | 48.7(28.2~66.4) | 40.9(26.6~59.7) | 48.2(32.2~98.5) |
| HOMA-IR | 1.74(1.19~2.77) | 1.99(1.19~2.99) | 2.22(1.56~3.04) | 1.83(1.11~2.85) | 1.88(1.09~2.99) | 2.00(1.55~2.85) | 1.87(1.20~2.90) | 2.12(1.03~2.93) | 1.91(1.25~2.87) | 1.77(0.80~3.08) |
| UACR | 14.6(6.5~34.6) | 20.9(10.9~102.6)* | 14.9(6.4~43.5) | 22.9(10.5~96.1) | 15.4(7.9~48.2) | 23.1(11.5~133.9) | 16.9(8.6~76.4) | 14.5(9.2~85.9) | 16.1(8.6~72.9) | 30.6(9.2~419.2) |
| OCHT（n=70） | | | | | | | | | | |
|  | ACEI/ARBs | | CCBs | | β-Blockers | | TDs | | α- Blockers | |
| Variables | No（23） | Yes（47） | No（14） | Yes（56） | No（46） | Yes（24） | No（60） | Yes（10） | No（65） | Yes（5） |
| HbA1c (%) | 9.63±1.95 | 9.23±2.55 | 9.59±2.82 | 9.30±2.26 | 9.04±2.13 | 9.98±2.69 | 9.00±2.08 | 11.53±2.90* | 9.42±2.37 | 8.58±2.40 |
| FBG (mmol/L) | 9.61±3.28 | 8.38±2.31 | 8.41±2.49 | 8.87±2.77 | 8.71±2.47 | 8.93±3.16 | 8.86±2.81 | 8.32±1.99 | 8.83±2.77 | 8.17±1.56 |
| AII (ng/L) | 112(106~128) | 114(106~123) | 113(103~122) | 112(106~125) | 114(106~124) | 112(106~124) | 113(106~123) | 113(102~129) | 112(106~122) | 132(107~144) |
| ALD (ng/L) | 151(127~184) | 147(110~182) | 158(99~193) | 144(116~181) | 138(110~179) | 155(122~185) | 144(110~180) | 158(131~235) | 147(116~183) | 132(102~319) |
| REN (ng/L) | 12.0(5.4~21.7) | 12.6(6.2~30.0) | 17.7(9.8~35.4) | 12.0(5.6~25.1) | 16.6(6.6~30.3) | 11.1(3.8~15.4) * | 12.3(5.4~26.6) | 16.5(8.6~28.4) | 12.6(5.7~26.5) | 12.6(6.9~31.4) |
| ARR | 1.20(0.63~2.76) | 1.05(0.60~2.11) | 0.93(0.59~1.62) | 1.19(0.65~2.70) | 1.00(0.59~1.81) | 1.55(0.99~3.51) * | 1.12(0.61~2.69) | 0.99(0.63~1.66) | 1.10(0.62~2.41) | 1.50(0.72~2.96) |
| CP (nmol/L) | 0.67 (0.43~0.90) | 0.57(0.34~0.88) | 0.83(0.43~1.08) | 0.56(0.35~0.86) | 0.54(0.36~0.87) | 0.66(0.41~1.01) | 0.60(0.35~0.90) | 0.74(0.43~1.31) | 0.63(0.38~0.90) | 0.40(0.33~1.45) |
| HOMA-β (%) | 44.5(23.6~68.3) | 42.2(28.2~69.1) | 58.0(36.5~80.1) | 41.9(27.9~67.6) | 41.9(29.7~63.7) | 47.3(27.0~105.4) | 41.9(28.1~68.9) | 52.7(40.3~81.8) | 44.5(28.0~68.7) | 36.5(33.2~85.1) |
| HOMA-IR | 1.81 (1.11~2.67) | 1.50 (0.90~2.27) | 2.11(1.13~3.21) | 1.50(0.98~2.27) | 1.39(0.98~2.28) | 1.75(1.27~2.76) | 1.57(1.00~2.29) | 1.92(1.07~3.52) | 1.66(1.03~2.35) | 0.99(0.82~3.78) |
| UACR | 14.1(6.2~23.1) | 12.9(7.4~27.4) | 8.5(6.1~14.90) | 13.9(8.1~28.5) | 11.8(6.2~20.4) | 15.9(10.3~27.8) | 13.0(6.3~24.6) | 12.7(9.7~24.5) | 12.3(6.4~18.9) | 29.8(22.4~270.4)* |
| DN（n=31） | | | | | | | | | | |
| Variables | ACEI/ARBs | | CCBs | | β-Blockers | | TDs | | α- Blockers | |
|  | No（4） | Yes（27） | No（5） | Yes（26） | No（19） | Yes（12） | No（25） | Yes（6） | No（29） | Yes（2） |
| HbA1c (%) | 10.50±2.61 | 9.32±1.92 | 10.66±2.04 | 9.24±1.96 | 9.88±2.02 | 8.82±1.89 | 9.77±1.98 | 8.22±1.74 | 9.59±1.98 | 7.67±2.08 |
| FBG (mmol/L) | 12.69±3.02 | 9.93±3.06 | 10.50±4.02 | 10.24±3.05 | 10.71±3.45 | 9.61±2.60 | 10.57±3.42 | 9.09±1.20 | 10.45±3.19 | 7.97±1.16 |
| AII (ng/L) | 119(95~177) | 122(113~127) | 124(116~129) | 121(106~130) | 120(106~127) | 126(114~160) | 120(109~130) | 126(108~137) | 122(113~127) | 115(101~129) |
| ALD (ng/L) | 136(79~200) | 155(110~192) | 174(121~231) | 146(103~192) | 172(120~205) | 127(86~159) | 155(113~198) | 135(99~187) | 155(110~192) | 276(105~448) |
| REN (ng/L) | 5.7(1.6~12.1) | 8.4(6.0~16.6) | 15.3(7.5~48.3) | 8.2(4.0~14.5) | 12.9(7.6~24.9) | 5.3(3.5~7.9) * | 8.6(6.2~16.1) | 5.2(3.8~20.0) | 8.0(4.7~15.3) | 66.8(8.9~124.8) |
| ARR | 3.44(1.58~4.94) | 1.53(0.86~2.35) | 0.90(0.55~1.89) | 1.92(1.06~3.46) | 1.18(0.77~2.13) | 2.26(1.91~3.99) * | 1.53(0.88~2.23) | 3.42(0.98~4.13) | 1.90(0.97~3.20) | 0.77(0.36~1.18) |
| CP (nmol/L) | 0.37(0.32~0.55) | 0.70(0.57~0.97) * | 0.62(0.39~0.84) | 0.68(0.49~1.00) | 0.62(0.47~0.97) | 0.80(0.58~1.06) | 0.62(0.48~0.95) | 0.86(0.66~1.15) | 0.66(0.56~0.92) | 0.86(0.28~1.44) |
| HOMA-β (%) | 17.8(9.7~27.6) | 45.6(23.1~55.7) * | 29.1(22.3~48.3) | 37.0(19.6~58.7) | 29.1(16.1~52.4) | 41.3(22.0~54.3) | 29.1(17.7~50.4) | 49.3(33.6~71.0) | 36.9(19.7~50.6) | 67.2(23.1~111.4) |
| HOMA-IR | 1.18(1.07~1.62) | 2.12(1.65~3.00) * | 1.63(1.04~2.87) | 1.96(1.57~2.77) | 1.70(1.33~2.69) | 2.16(1.77~2.88) | 1.75(1.46~2.67) | 2.22(1.78~3.12) | 1.91(1.63~2.65) | 2.14(0.74~3.53) |
| UACR | 130(51~10011) | 146(71~979) | 108(79~241.8) | 163(40~1270) | 137(71~811) | 159(45~1881) | 137(72~1113) | 361(35~1240) | 137(71~926) | 899(551~1247) |

Data were expressed as“mean ± standard deviation” or “median with interquartile range”. HT, hypertension; DN, diabetic nephropathy; OCHT, other diabetic complications in hypertensive patients. HbA1c, hemoglobin A1c; FBG, fast blood glucose; AII, Angiotensin II; ALD, aldosterone; REN, renin; ARR, aldosterone-to-renin ratio; CP, C-peptide; HOMA-β, Homeostatic Model Assessment of β-cell function; HOMA-IR, Homeostatic Model Assessment of insulin resistance; UACR, urinary albumin-to-creatinine ratio. (**p*<0.05)
