# Supplementary material for: Renin-angiotensin-aldosterone system variations in type 2 diabetes mellitus patients with different complications and treatments: Implications for glucose metabolism
Source: PLoS One. 2025 Mar 19;20(3):e0316049. doi: 10.1371/journal.pone.0316049 (PMC11922211; doi:10.1371/journal.pone.0316049)
Supplement: S3 Table — (DOCX) [file pone.0316049.s003.docx]

S3 Table. Antidiabetic treatment of 191 T2DM patients with hypertension or normotension at different subgroups.

| Subgroups | Metformin | α-glycosidase inhibitors | Insulin | SGLT-2is | GLP-1RAs | DPP-4is | TZDs | Sulfonylureas | Glinides |
| --- | --- | --- | --- | --- | --- | --- | --- | --- | --- |
| DMHT NO. (%) | | | | | | | | | |
| DN(n=31) | 22(71.0) | 24(77.4) | 24(77.4) | 17(54.8) | 4(12.9) | 11(35.5) | 6(19.4) | 1(3.2) | 4(12.9) |
| DK(n=20) | 18(90.0) | 12(60.0) | 18(90.0) | 9(45.0) | 9(45.0) | 5(25.0) | 12(60.0) | 2(10.0) | 1(5.0) |
| DNK(n=10) | 8(80.0) | 8(80.0) | 7(70.0) | 5(50.0) | 5(50.0) | 3(30.0) | 3(30.0) | 1(10.0) | 1(10.0) |
| OCHT(n=70) | 56(80.0) | 57(81.4) | 48(68.6) | 32(45.7) | 12(17.1) | 18(25.7) | 23(32.9) | 6(8.6) | 3(4.3) |
| NCHT(n=20) | 16(80.0) | 14(70.0) | 14(70.0) | 11(55.0) | 4(20.0) | 5(25.0) | 11(55.0) | 1(5.0) | 2(10.0) |
| Total(n=151) | 120(79.5) | 115(76.2) | 111(73.5) | 74(49.0) | 34(22.5) | 42(27.8) | 55(36.4) | 11(7.3) | 11(7.3) |
| DMNT NO. (%) | | | | | | | | | |
| OCNT(n=20) | 16(80.0) | 14(70.0) | 15(75.0) | 11(55.0) | 3(15.0) | 7(35.0) | 7(35.0) | 1(5.0) | 2(10.0) |
| NCNT(n=20) | 15(75.0) | 12(60.0) | 14(70.0) | 10(50.0) | 3(15.0) | 6(30.0) | 8(40.0) | 2(10.0) | 1(5.0) |
| Total(n=40) | 31(77.5) | 26(65.0) | 29(72.5) | 21(52.5) | 6(15.0) | 13(32.5) | 15(37.5) | 3(7.5) | 3(7.5) |

DMHT, diabetes mellitus patients with hypertension; DMNT, diabetes mellitus patients with normotension; DN, diabetic nephropathy; DK, diabetic ketoacidosis; DNK, diabetic nephropathy with ketoacidosis; OCHT, other diabetic complications in hypertensive patients; NCHT, no complications in hypertensive patients; OCNT, other diabetic complications in normotensive patients; NCNT, no complications in normotensive patients;  SGLT-2is, sodium-glucose cotransporter-2 inhibitors; GLP-1RAs, glucagon-like peptide-1 receptor agonists; DPP-4is, dipeptidyl peptidase-4 inhibitors; TZDs, thiazolidinediones; NO., number.
