# Supplementary material for: Renin-angiotensin-aldosterone system variations in type 2 diabetes mellitus patients with different complications and treatments: Implications for glucose metabolism
Source: PLoS One. 2025 Mar 19;20(3):e0316049. doi: 10.1371/journal.pone.0316049 (PMC11922211; doi:10.1371/journal.pone.0316049)
Supplement: S4 Table — (DOCX) [file pone.0316049.s004.docx]

S4 Table. The influence of antidiabetic therapy on the RAAS system.

| DMHT(n=151) | | | | | |
| --- | --- | --- | --- | --- | --- |
| Anti-diabetic Agents (NO.) | | AII (ng/L) | ALD (ng/L) | REN (ng/L) | ARR |
| Metformin | No (31) | 110(105~124) | 137(97~236) | 11.3(6.2~24.6) | 1.25(0.63~2.16) |
|  | Yes (120) | 116(106~126) | 151(119~188) | 12.6(6.0~26.6) | 1.18(0.61~2.25) |
| α-glycosidase inhibitors | No (36) | 113(103~122) | 141(111~209) | 9.7(4.8~25.7) | 1.36(0.72~2.53) |
|  | Yes (115) | 116(107~127) | 150(116~184) | 12.9(6.5~27.0) | 1.19(0.60~2.16) |
| Insulin | No (40) | 116(103~125) | 158(122~202) | 14.6(6.0~29.7) | 1.05(0.58~2.52) |
|  | Yes (111) | 115(106~126) | 147(114~189) | 11.5(6.2~25.9) | 1.26(0.63~2.16) |
| SGLT-2is | No (77) | 115(106~126) | 150(124~210) | 11.7(5.8~26.8) | 1.25(0.60~2.58) |
|  | Yes (74) | 115(106~125) | 148(111~184) | 13.0(6.3~25.6) | 1.14(0.67~2.11) |
| GLP-1RAs | No (117) | 116(106~127) | 151(116~196) | 12.6(6.3~25.2) | 1.20(0.69~2.16) |
|  | Yes (34) | 114(104~124) | 139(120~172) | 10.8(5.4~28.7) | 1.06(0.50~2.80) |
| DPP-4is | No (109) | 113(105~127) | 150(123~195) | 12.0(6.0~26.1) | 1.26(0.69~2.16) |
|  | Yes (42) | 118(110~122) | 142(101~182) | 12.1(6.2~31.5) | 1.11(0.59~2.29) |
| TZDs | No (96) | 116(107~127) | 147(117~188) | 11.5(6.0~21.6) | 1.35(0.70~2.15) |
|  | Yes (55) | 113(100~124) | 156(114~192) | 14.0(6.3~28.8) | 1.05(0.57~2.30) |
| Sulfonylureas | No (140) | 115(106~126) | 150(116~192) | 12.6(6.0~26.9) | 1.20(0.61~2.30) |
|  | Yes (11) | 115(104~124) | 143(127~167) | 11.5(6.6~13.6) | 1.10(0.87~1.48) |
| Glinides | No (140) | 115(106~125) | 150(119~192) | 12.6(6.2~26.3) | 1.18(0.61~2.16) |
|  | Yes (11) | 119(107~156) | 137(97~170) | 8.4(4.7~27.0) | 1.80(0.83~2.70) |
| DMNT(n=40) | | | | | |
| Anti-diabetic Agents (NO.) | | AII (ng/L) | ALD (ng/L) | REN (ng/L) | ARR |
| Metformin | No (9) | 103(97~138) | 120(98~148) | 8.9(6.3~20.9) | 0.90(0.73~2.16) |
|  | Yes (31) | 101(90~110) | 117(97~167) | 8.9(4.5~23.8) | 0.99(0.75~2.57) |
| α-glycosidase inhibitors | No (14) | 106(97~115) | 127(96~213) | 12.7(5.5~25.9) | 0.93(0.76~1.87) |
|  | Yes (26) | 100(94~104) | 116(98~146) | 8.5(4.5~19.1) | 1.65(0.73~2.52) |
| Insulin | No (11) | 103(99~113) | 130(98~182) | 8.0(5.5~25.0) | 1.46(0.76~2.15) |
|  | Yes (29) | 100(93~111) | 115(96~146) | 9.0(4.5~21.8) | 0.87(0.73~2.50) |
| SGLT-2is | No (19) | 101(95~110) | 130(100~178) | 8.0(5.3~24.0) | 1.78(0.75~2.57) |
|  | Yes (21) | 101(94~120) | 115(92~137) | 8.9(4.6~20.9) | 0.96(0.74~2.25) |
| GLP-1RAs | No (34) | 101(90~111) | 116(97~151) | 9.0(4.6~22.9) | 0.93(0.75~2.50) |
|  | Yes (6) | 104(97~127) | 126(106~158) | 7.7(5.3~25.0) | 1.81(0.61~2.46) |
| DPP-4is | No (27) | 102(90~111) | 117(98~150) | 8.7(4.5~22.6) | 1.48(0.62~2.71) |
|  | Yes (13) | 100(97~113) | 123(91~178) | 9.0(6.8~20.7) | 0.90(0.81~1.80) |
| TZDs | No (25) | 100(93~108) | 115(96~150) | 8.0(4.5~19.1) | 1.48(0.78~2.50) |
|  | Yes (15) | 102(98~120) | 123(98~178) | 16.5(5.5~24.0) | 0.87(0.60~2.15) |
| Sulfonylureas | No (37) | 101(93~112) | 117(97~148) | 8.9(4.6~21.8) | 0.99(0.74~2.43) |
|  | Yes (3) | 104(102~108) | 167(134~194) | 13.2(9.9~21.0) | 0.76(0.76~1.67) |
| Glinides | No (37) | 102(96~113) | 120(98~152) | 8.9(4.6~23.2) | 0.99(0.74~2.50) |
|  | Yes (3) | 99(95~99) | 98(85~114) | 8.0(6.8~12.7) | 0.90(0.83~1.34) |

DMHT, diabetes mellitus patients with hypertension; DMNT, diabetes mellitus patients with normotension; AII, angiotensin II; ALD, aldosterone; REN, renin; ARR, aldosterone to renin ratio; SGLT-2is, sodium-glucose cotransporter-2 inhibitors; GLP-1RAs, glucagon-like peptide-1 receptor agonists; DPP-4is, dipeptidyl peptidase-4 inhibitors; TZDs, thiazolidinediones; NO., number.
