# Supplementary figures and images for: Renin-angiotensin-aldosterone system variations in type 2 diabetes mellitus patients with different complications and treatments: Implications for glucose metabolism
Source: PLoS One. 2025 Mar 19;20(3):e0316049. doi: 10.1371/journal.pone.0316049 (PMC11922211; doi:10.1371/journal.pone.0316049)

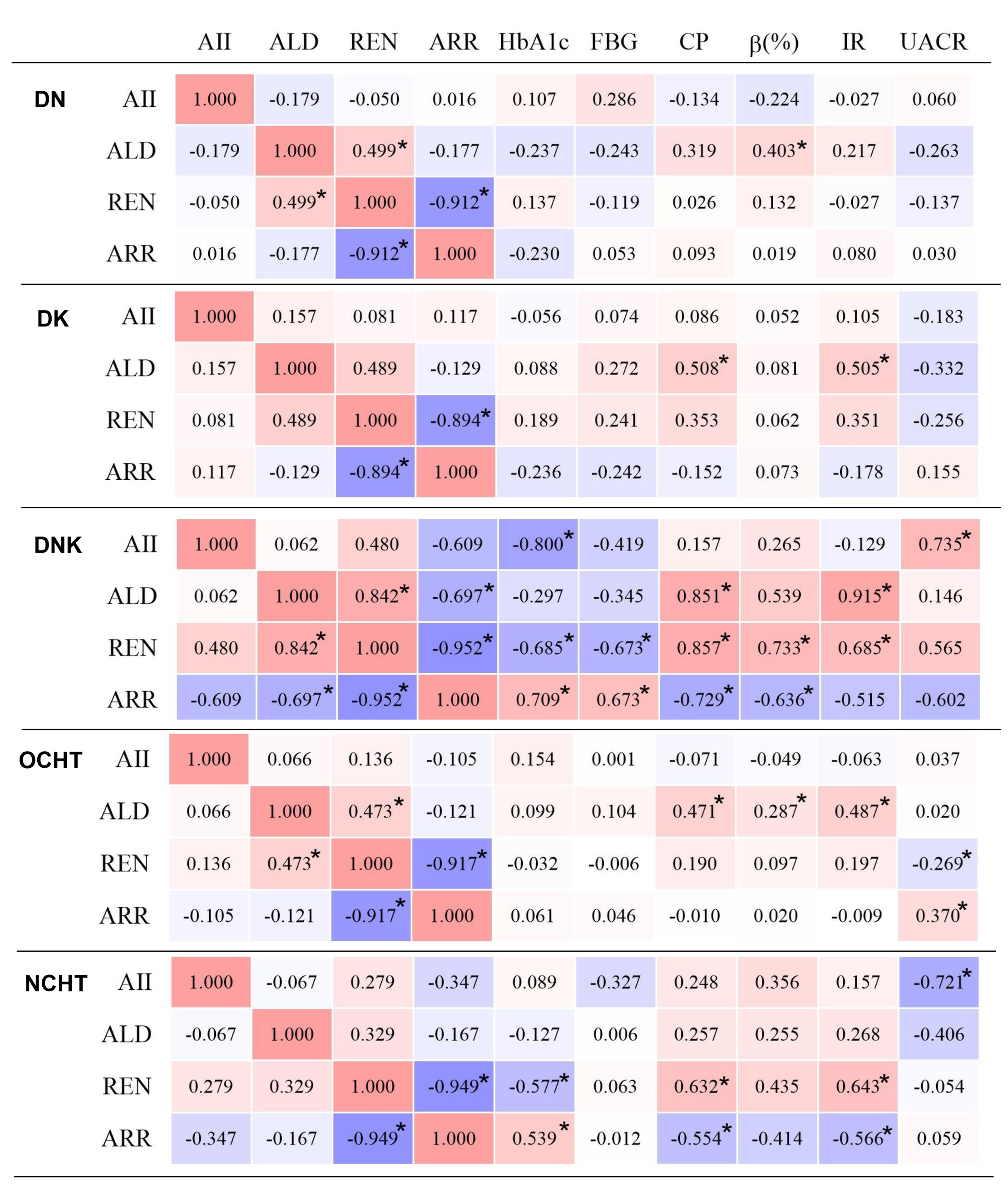

Supplement: S1 Fig — Heat-map with background in three-color scale where − 1 = blue, + 1 = red and 0 = white. DN, diabetic nephropathy; DK, diabetic ketoacidosis; DNK, diabetic nephropathy with ketoacidosis; OCHT, other diabetic complications in hypertensive patients; NCHT, no complications in hypertensive patients; AII, Angiotensin II; ALD, aldosterone; REN, renin; ARR, aldosterone-to-renin ratio; HbA1c, hemoglobin A1c; FBG, fast blood glucose; CP, C-peptide; β (%), Homeostatic Model Assessment of β-cell function; IR, Homeostatic Model Assessment of Insulin Resistance; UACR, urinary albumin-to-creatinine ratio. (*p < 0.05). (TIF) [file pone.0316049.s005.tif]

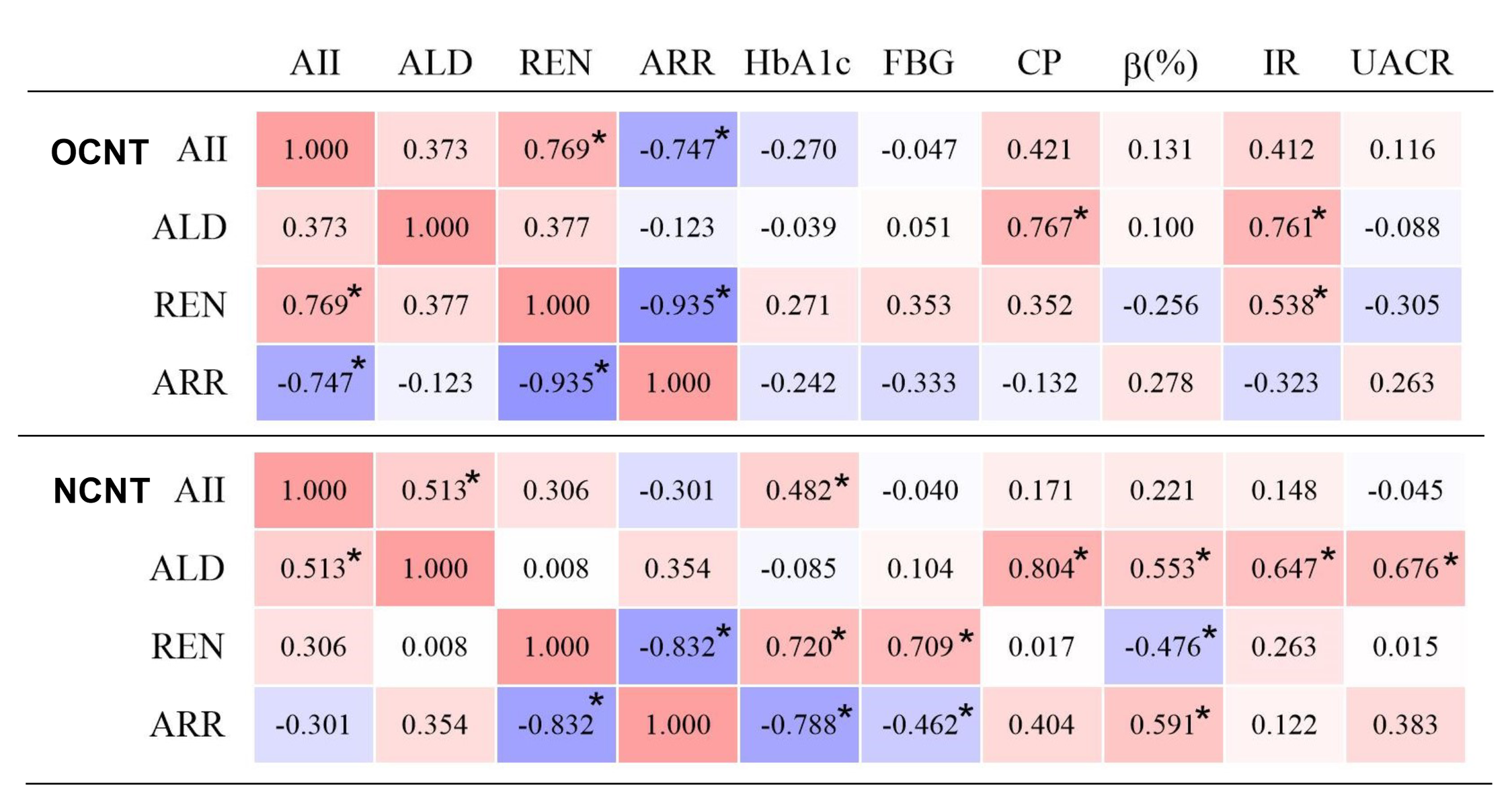

Supplement: S2 Fig — Heat-map with background in three-color scale where − 1 = blue, + 1 = red and 0 = white. OCNT, other diabetic complications in normotensive patients; NCNT, no complications in normotensive patients; AII, Angiotensin II; ALD, aldosterone; REN, renin; ARR, aldosterone-to-renin ratio; HbA1c, hemoglobin A1c; FBG, fast blood glucose; CP, C-peptide; β (%), Homeostatic Model Assessment of β-cell function; IR, Homeostatic Model Assessment of Insulin Resistance; UACR, urinary albumin-to-creatinine ratio. (*p < 0.05). (TIF) [file pone.0316049.s006.tif]
